# Reframing individual roles in collaboration: digital identity construction and adaptive mechanisms for resistance-based professional skills in AI-human intelligence symbiosis

**DOI:** 10.3389/fpsyg.2025.1652130

**Published:** 2025-08-08

**Authors:** Weizheng Jiang, Yongzhou Li, Xiaoling Hu, Dongling Ma

**Affiliations:** ^1^School of Management, Wuhan University of Science and Technology, Wuhan, China; ^2^School of Management, Wuhan Technology and Business University, Wuhan, China

**Keywords:** digital identity construction, AI-resistant professional skills, AI-HI collaborative innovation performance, knowledge articulation, collaborative intelligence

## Abstract

Amid the unprecedented wave of AI advancement, AI-resistant professional skills play a significant role in enhancing the effectiveness of human–AI collaboration. However, existing research tends to isolate professional skills from their broader context, overlooking the triadic construction of digital identity recognition through individual motivation, structural position, and knowledge articulation. This oversight weakens the sustainability and adaptability of skill expression, thereby hindering innovation performance in AI–HI (Artificial Intelligence–Human Intelligence) collaboration. Drawing on the entropy weight method, gradient descent algorithm, and a residual–matching decision matrix, this study conducted quantitative modeling of 418 participants in the financial co-production sector from 2022 to 2024. The findings reveal that network centrality (NC; β = 0.04^**^) and proactive personality (PP; β = 0.05^**^) significantly amplify the impact of two key AI-resistant skills—foreign language proficiency (FL) and passion/optimism (PO)—on collaboration effectiveness, through structural empowerment and intrinsic motivation. Furthermore, this study develops a digital identity recognition and classification framework that identifies three distinct groups: core innovators, marginal experts, and low performers. By extending the theoretical model of digital identity construction within AI–HI collaboration, this study also proposes a differentiated approach to talent development and resource allocation based on innovation effectiveness and identity alignment, offering new insights into the advancement of digital human capital.

## 1 Introduction

Since 2022, the development of generative AI (GenAI) has accelerated at an extraordinary pace, continuously deepening its integration and entanglement with a wide range of industries. However, evidences have emerged pointing to a set of global challenges accompanying AI's advancement: the remarkable acceleration in technological development has not been matched by a corresponding increase in collaborative effectiveness. According to the *AI Index Report 2024* (Clark et al., 2024), investments related to GenAI surged 8fold in 2023, reaching USD 25.2 billion. In specific tasks such as image classification and language comprehension, AI has already surpassed human performance. In a striking contrast, the report also reveals that in complex tasks—such as visual reasoning, mathematical reasoning, and creative problem-solving—AI has not demonstrated a clear advantage.

Furthermore, McKinsey's *The State of AI* report (Singla et al., 2025) points to a series of organizational challenges faced by companies embedding AI into their business processes: misalignment between deployment workflows and human resource structures, difficulties in hiring for AI-related roles, underdeveloped governance and trust mechanisms, and the need for a fundamental reconfiguration of AI-HI interaction. Collectively, these conditions underscore the necessity of AI-human symbiosis, while also revealing that effective mechanisms for AI-HI collaboration have yet to be fully established.

Echoing this global concern, China is likewise experiencing a “high technology-low collaboration” paradox. According to the CAICT (2024), China's core AI industry grew by 13.9% year-on-year in 2023, and by the first quarter of 2024, a total of 478 large-scale AI models had been released. However, data from the iResearch Group (2024) indicates that the growth of China's collaborative office platforms has slowed significantly since 2022 and has even begun to decline. This structural tension between rapid technological advancement and declining collaboration raises fundamental questions about AI-HI interaction: does the acceleration of AI development necessarily lead to collaborative innovation at the organizational level? Or does the disjunction between speed and effectiveness reflect a deeper disruption—one in which AI challenges not only the traditional roles of human collaboration but also the cognitive mechanisms underlying them? In such an AI-human symbiotic environment, must humans seek new forms of identity and positioning?

This dilemma is particularly evident in practical applications. For example, Zoom's *AI Companion*, regarded as a benchmark for remote collaboration, achieves only about a 50% success rate in generating accurate meeting summaries. Although generative AI (GenAI) possesses high programmability and strong logical capabilities, the absence of mechanisms for “role construction” and “digital identity adaptation” often results in information silos, unclear responsibility boundaries, and impeded collaboration (Okamura and Yamada, 2020). Moreover, the challenge of task allocation between humans and AI is deeply shaped by the characteristics of the AI system itself. A lack of transparency or limited perceptibility can directly undermine trust mechanisms in human–AI interactions (Rai, 2020). In addition, low levels of anthropomorphism in AI often result in an absence of emotional support (Sheng et al., 2024). Overlooking these system-level factors can lead to role misalignment and psychological dissonance within collaborative human–AI settings.

These findings prompt a rethinking of the practical challenges in AI-HI collaborative innovation. By approaching the issue through the lens of individual digital identity construction, this study identifies mismatch patterns in identity recognition that affect collaboration performance and proposes a mechanism to optimize identity-role alignment.

Existing research widely emphasizes the value of AI in collaborative innovation, highlighting its advantages in prediction, efficiency, and knowledge acquisition (Chu et al., 2023). However, much of the literature remains trapped in a binary opposition between technological determinism and capability determinism: the former overemphasizes the direct impact of technological features, while the latter treats professional skills in isolation from their broader context. Both perspectives neglect the complexity inherent in establishing a symbiotic relationship between AI-HI. As illustrated in McKinsey's *What Employees Are Saying About the Future of Remote Work* report (Andrea et al., 2021), factors such as social network construction and psychological needs play a critical role in digital workplace collaboration. In fact, there is a complex and dynamic interplay among individual intrinsic motivations, social network positions, knowledge articulation, and AI-HI role allocation (Alowais et al., 2023; Jia et al., 2024). Only by considering and modeling these elements holistically can we effectively uncover the true mechanisms underlying AI-HI collaborative innovation.

Building on the above background, this study seeks to address a critical question: how do professional skills, coexisting with AI, influence collaborative innovation in multifaceted environments? To this end, we adopt a triadic synergy perspective that emphasizes the interaction among individual personality traits, the distribution of professional skills, and social network construction, and develop a quantitative framework for analyzing AI-HI collaborative innovation.

However, existing research on AI-driven professional skills exhibits three main limitations. First, many studies focus solely on the distribution of professional skills within industries, overlooking the actual impact of these skills on AI-enabled innovation performance (e.g., Alekseeva et al., 2021; Fletcher and Thornton, 2023). Second, few investigations integrate personal traits and social networks, resulting in overfitting issues between skills and behavioral effects. For instance, Chuang (2024) argues that the full potential of AI technologies depends on collective intelligence. Third, social network position disrupts the pathways of professional skill conversion. This is especially true for soft skills, whose effectiveness often hinges on informal networks—an aspect insufficiently addressed in current literature (Cangialosi et al., 2021; e.g., Burtch et al., 2023).

From a practical perspective, existing research remains insufficient in decomposing and prioritizing AI-related professional skills. Quantitative assessments of such skills are largely based on self-reported questionnaires, which lack objectivity. Hard skills, being observable and recordable capabilities, should be measured through more rigorous and objective approaches (i.e., Alekseeva et al., 2021). Moreover, the extent to which professional skills are translated into collaborative innovation performance should not be inferred solely from subjective scales (e.g., Cangialosi et al., 2021; Chowdhury et al., 2022; Pham et al., 2024); instead, they should be evaluated in relation to the actual task allocation between humans and AI within the collaboration process.

To address these limitations and research gaps, we implemented the following measures. First, we incorporated a triadic synergy framework to investigate the interaction among personality traits, social network structure, and professional skill expression in AI-HI collaborative innovation. Second, we adopted the After Action Review (AAR) method to objectively capture cognitive performance outcomes and assess the effectiveness of AI-HI collaboration, particularly with regard to hard skills. Third, we applied Gradient Descent (GD) techniques to explore the distribution of AI-driven professional skills, rather than treating soft and hard skills as monolithic constructs, thereby improving the sensitivity of measurement and prioritization. Fourth, we constructed a residual- matching analytical matrix to reconceptualize digital identity from an interactional perspective and proposed targeted knowledge management strategies accordingly.

This study surveyed 418 participants from AI-enabled co-production industries in the financial sector between 2022 and 2024. Data were collected on proactive personality (PP) traits, community network structures, and cognitive skills to uncover the mechanisms through which AI-driven professional skills shape AI-HI role allocation. These findings offer new insights into the formation and development of digital human capital theory.

## 2 Theoretical framework and research questions

### 2.1 Digital identity construction in triadic synergy

#### 2.1.1 The definition of digital identity and essential dimensions

The construction of digital identity spans multiple disciplines, including information technology, sociology, and psychology. Its formation is dynamic, continuous, and multilayered. According to Sedlmeir et al. (2021), digital identity refers to the representation of an entity within a virtual environment—an entity that may include not only individual humans but also legal persons or technological agents. A digital identity is composed of controllable attributes (such as behavioral records, technical features, and social parameters) and is anchored by a unique identifier that ensures consistency across platforms. This definition emphasizes both the sustainability (recognizability) and the diversity of identity features. Furthermore, digital identity is co-constructed by individuals, organizations, and technological systems. With the emergence of decentralized management approaches, identity assignment is increasingly distributed and individually governed, granting users greater autonomy and digital sovereignty.

Based on the definition and connotation of digital identity, this study proposes a triadic synergy mechanism composed of intrinsic motivation, structural positioning, and knowledge articulation. This framework aims to capture the generative logic of digital identity in an AI-HI symbiotic environment. The construction of such identity is not only critical to the allocation of roles in AI-HI collaboration but also plays a key role in the sustainable adaptation of knowledge co-creation and innovation.

Digital identity within the triadic synergy framework comprises three essential dimensions:

##### 2.1.1.1 Knowledge articulation

Represented by quantifiable professional skills, this dimension reflects the extent to which individuals can mobilize their intrinsic motivations and leverage structural positions in AI-HI collaboration (e.g., Chuang, 2024). Collaborative intelligence emphasizes the complementarity, reciprocity, and co-evolution between humans and AI. It reflects not only the technical challenges of embedding AI into work processes, but also the alignment and synergy between AI systems and individual knowledge articulation (Tariq et al., 2025).

##### 2.1.1.2 Structural positioning

Measured through social network centrality, this captures the external visibility and structural opportunities of one's identity on digital platforms (i.e., Morrison-Smith and Ruiz, 2020). This indicator directly pertains to hybrid teaming frameworks—specifically, how structural position advantages can be leveraged to integrate human creativity and emotional intelligence with the strengths of AI, thereby forming a highly efficient and adaptable collaborative unit (Caldwell et al., 2022).

##### 2.1.1.3 Intrinsic motivation

Exemplified by PP traits, this dimension provides the motivational foundation of digital identity. In AI-HI collaboration, self-determination positively influences exploratory activities in AI-HI symbiosis (e.g., Kong et al., 2024). When AI is designed as human-centered (or HCAI) and collaboration-enhancing, individuals are more likely to exhibit proactive agency rather than passive acquiescence or substitution anxiety (Shneiderman, 2022).

#### 2.1.2 Knowledge articulation: professional skills

##### 2.1.2.1 Hard skills in AI-HI collaboration

According to Hendarman and Cantner (2018), hard skills refer to professional knowledge and technical competencies that can be described, quantified, preserved, and documented, and these sets of competencies are relevant to specific tasks. The content of hard skills varies across professional fields, but the vast majority of hard skill sets involve computer competence and digital literacy, as investigated by Alekseeva et al. (2021), and Chuang (2024).

In the integration of computer and AI technologies, computer operations, machine learning understanding, and programming involve the expression of computer and network technologies in individual behavior, i.e. Cyber Behavior (CB). Analysis, modeling, and mathematical foundations affect the individual's access to the laws behind the data, i.e., Date Analysis (DA). They play a key role in the understanding and application of AI. Not only do they reflect learners' mastery of superficial AI skills, but also demonstrate their understanding and insight into the principles and mechanisms behind the operation of AI, as Bankins et al. (2024) conclude that algorithms and human capabilities influence employees' own experience and job design.

However, the differential impact from hard skills is not only reflected in job and innovation performance, but also in the regional adaptation to digital technologies, as investigated by Carlisle et al. (2023), who argues that CB and DA are far less important than communication skills in the digital transformation of the service sector. Whereas, mastery of a Foreign Language (FL) has been considered as an important hard skill in many studies, functioning to facilitate cross-cultural communication and knowledge flow (Zeng and Yang, 2024).

##### 2.1.2.2 Soft skills in AI-HI collaboration

According to Hendarman and Cantner (2018) summary, soft skills involve relational resources and communication skills aimed at environmental adaptation through interpersonal embedding and are informal skills. In contrast to hard skills, the prominent role of soft skills lies in the development of communication skills, teamwork and problem solving skills. For human-computer coexistence relationships, these competences are not only necessary for the modern workplace (Escolà-Gascón and Gallifa, 2022). Their acquisition and enhancement can also increase human-AI trust, playing a key role in the maintenance of friendly AI-HI coexistence relationships (Sheng et al., 2024).

Soft skills can be expressed as Innovation Leadership (IL), Relationship Building (RB), Tolerance for Uncertainty (TU), and Passion and Optimism (PO) (2018). Soft skills likewise drive digital transformation and demonstrate criticality in AI-driven collaborative innovation. IL is related to technology acceptance management, which influences collaboration across disciplines and domains (Bahoo et al., 2023). RB competencies derive from field theory and group dynamics, which are beneficial for the building of hybrid AI social networks (Ng, 2022). Leadership types holding TUs are beneficial for promoting psychological safety and facilitating incremental innovation (Uhl-Bien, 2021), and PO sustains positive individual perceptions of change and coexistence in the AI era (Burtch et al., 2023).

Therefore, as a crucial component of the knowledge articulation, professional skills carry the dual function of task execution and knowledge exchange within AI–HI collaboration. Hard skills reflect an individual's depth of understanding and operational proficiency with AI technologies, while soft skills signify the capacity to collaborate effectively, enhance communication, and foster trust. Together, these skill sets shape the visibility, reliability, and role recognition of individuals in the process of digital identity construction.

#### 2.1.3 Structural position: network centrality

Social capital is manifested through structural positions within social networks, with centrality (including relative centrality, betweenness centrality, etc.) being a key indicator of social capital. Individuals occupying central positions, with higher information flow efficiency and influence, can more effectively promote knowledge spillover and innovation (Cangialosi et al., 2021).

Building on this foundation, hybrid AI social networks not only optimize information transmission pathways and strengthen relational connections. These enhancing individuals' structural embeddedness, but also amplify the leverage effect of personal centrality on knowledge collaboration through augmented social computing. As Wang et al. (2022) argue, H-AI (hybrid human–AI) systems, as an augmented intelligence paradigm, are essential in addressing the limitations of conventional AI when confronting complex and dynamic social problems. This perspective indirectly highlights the foundational role that network structural advantages play in the construction of social behavior within digital identity formation.

From the perspective of hard skills, digital capability gaps often lead to issues in task completion time and efficiency. These gaps are heavily influenced by one's structural position within the social network. Individuals at the core of the network typically have greater access to information and communication opportunities, which in turn accelerates the expression and application of hard skills (Lythreatis et al., 2022).

Soft skills can be expressed as communication abilities, teamwork capabilities, adaptability, and emotional intelligence. These skills are integral to the maintenance and establishment of centrality. Furthermore, while virtual teams and remote collaboration rely on technology, communication and trust remain essential. Particularly in virtual networks, self-organization can easily lead to disorderly project development. Therefore, a higher Network Centrality (NC) implies trust and commitment, which facilitates the correct and efficient operation of professional skills.

In summary, NC reflects two key aspects of an individual's structural position: the efficiency of information flow and knowledge collaboration, and the recognition of one's role within their digital identity. Within hybrid AI-enabled social networks, individuals with higher centrality are more likely to be identified as knowledge hubs. Such structural advantages enhance the external visibility and system-level recognition of their digital identity, making their role in AI–HI collaboration more stable and sustainable.

#### 2.1.4 Intrinsic motivation: proactive personality

Proactive Personality (PP), as a form of intrinsic motivation, is closely linked to self-determination. It is often studied in terms of how individuals activate themselves positively by constructing both internal and external sources of meaning. First, from the perspective of external relationship building, proactive individuals initiate positive changes in their interactions through internal drives, expanding their social networks and thereby increasing the likelihood of realizing personal visions (Rienda et al., 2025). Second, from the standpoint of internal meaning-making, individuals with high levels of proactivity tend to assign deeper significance to learning, work, and personal effort. This capacity enables them to more effectively translate intrinsic motivation into concrete expressions of creativity (Zhang et al., 2021).

From the perspective of technology acceptance theory, trust in AI is associated with dimensions such as transparency, functionality, reliability, explainability, and perceived benevolence (Huang and Rust, 2024). While transparency, explainability, and functionality relate to the design and performance of AI systems, the extent to which these features are perceived and trusted largely depends on users' willingness to engage and their cognitive orientation. Zheng et al. (2020), through a study on online learning during the COVID-19 pandemic, found that PP significantly enhanced the quality of online interaction, self-efficacy, and social capital. Our latest research also indicates that excessive AI transparency may hinder the development of higher-order cognition. Enhancing individual digital identity through collaborative intelligence depends on the cultivation of effective learning and self-management abilities (Jiang et al., 2025). These findings suggest that proactive individuals are more inclined to explore and engage with technology, which in turn improves their understanding of and trust in the complex mechanisms underlying AI—thus forming a psychological foundation for technology trust.

Therefore, as a driving force behind digital identity formation, a proactive “self-driven identity” not only facilitates skill expression and network embeddedness in AI–HI collaboration but also strengthens the trust relationship between humans and AI systems.

### 2.2 Limitations in measuring AI-HI collaborative innovation and cognitive dynamics

**Figure 1:**
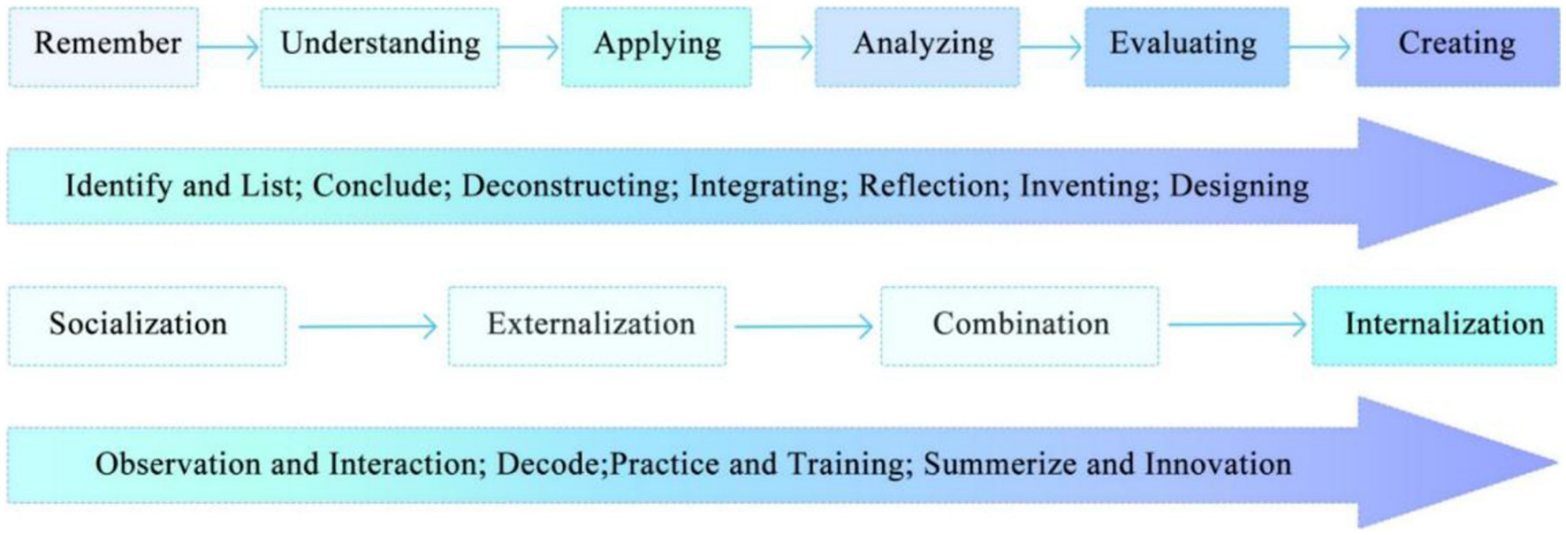
Correlation between SECI model and Bloom's Taxonomy.

The dynamic reorganization of cognitive resources within social networks is the essence of knowledge collaboration and innovation. Nonaka's (2022) early SECI model indirectly reflects the phased progression of cognitive strategies: observational imitation (socialization) → metaphorical encoding (externalization) → deconstruction and reconstruction (combination) → learning transfer (internalization). Bloom's taxonomy further explains the transformation of individual cognition in the learning process. By defining cognitive levels, Bloom's framework provides an operational theoretical label for quantifying knowledge collaboration and innovation. Logically, this framework aligns with the SECI knowledge spiral model (see Figure 1). The socialization stage, which relies on observational imitation, corresponds to Bloom's cognitive categories of remembering and understanding, while the combination stage, requiring deconstruction and reconstruction, aligns with application and analysis. These cognitive behaviors form the fundamental units of the innovation process, and the distribution of different cognitive levels within this process serves as an indicator of collaboration and innovation efficiency.

However, the staged division of cognitive levels remains ambiguous, and AI intervention is reshaping the division of cognitive labor in the following ways:

#### 2.2.1 AI as a substitute for lower-order cognition

AI automates a vast range of procedural and repetitive cognitive tasks, such as encoding, decoding, and information retrieval (corresponding to the externalization and combination stages), thereby creating favorable conditions for the acquisition of complex and breakthrough cognition (e.g., Hu et al., 2021).

#### 2.2.2 AI as an enhancer of higher-order cognition

While offloading large volumes of procedural and repetitive tasks, collaborative intelligence facilitates the transfer and conversion of complex tacit knowledge—thus accelerating internalization (Asrifan et al., 2024). At the same time, human-centered AI and generative technologies can effectively support decision-making and foster creativity (Shneiderman, 2022; Jia et al., 2024; Ritala et al., 2024).

Interestingly, this disruptive transformation reveals a dual deficiency in current quantitative research approaches:

#### 2.2.3 Measurement distortion

Current studies rely heavily on self-reported surveys (e.g., Pham et al., 2024), which fail to capture AI-induced variations in cognitive behavior, such as the flow and conversion of tacit knowledge in AI-HI collaboration. Consequently, they struggle to effectively distinguish AI-enabled collaborative innovation from traditional modes.

#### 2.2.4 Paradigmatic lag

Existing cognitive quantification research continues to conceptualize cognitive levels as a linear progression, neglecting the “cognitive leaps” and transformative shifts induced by AI. For instance, Bharatha et al. (2024) argue that ChatGPT functions as a complementary mechanism to human cognition in medical education, reinforcing the notion that AI fundamentally reshapes human cognitive processes.

Therefore, it is necessary to establish a new paradigm for cognitive quantification to reflect the efficiency driven by AI. Mainly through the After-Action Review (AAR; Keiser and Arthur, 2021), individual cognition is quantified, and the cognitive changes and reorganization during knowledge transformation are dynamically tracked (Nonaka and Yamaguchi, 2022; Asrifan et al., 2024) to reveal the laws of AI-HI collaboration.

### 2.3 Research questions

**Figure 2:**
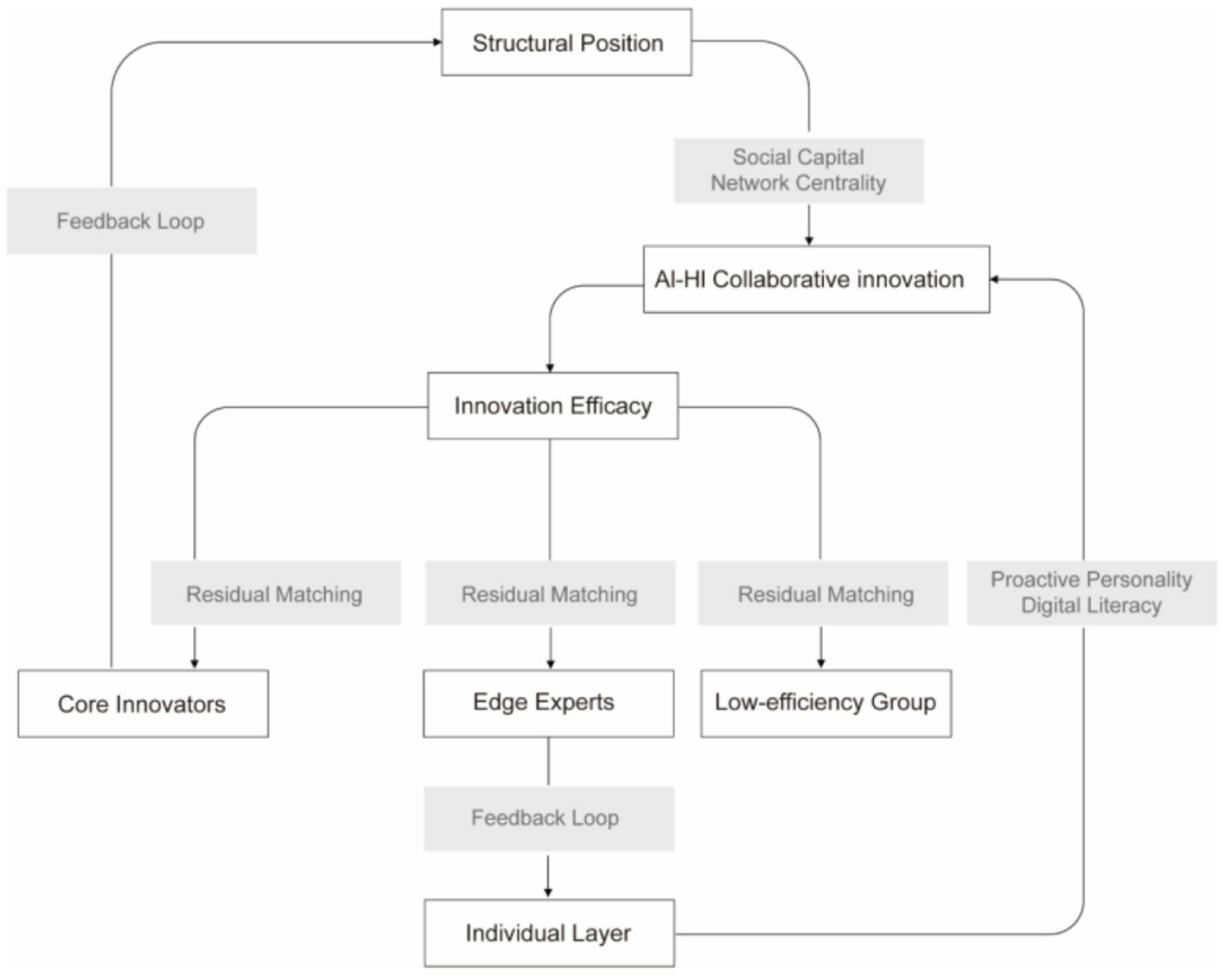
Research model and conceptual framework.

Building upon the triadic synergy framework, this study conceptualizes digital identity as the outcome of interactions among individual role cognition (i.e., PP), platform-based recognition (i.e., social network position), and knowledge articulation (i.e., professional skills) in AI-HI collaboration. An individual's identity is formed through the dynamic interplay of these three components, which together shape the cognitive impact of AI-HI collaboration. This dynamic mechanism and its effect on collaborative innovation effectiveness are illustrated in Figure 2.

Based on this framework, we pose the following research questions:

RQ1: Which professional skills demonstrate greater “AI resistance,” meaning they are more likely to consistently convey identity and knowledge value in collaborative settings?
RQ2: Within the AI-HI symbiotic relationship, can the triadic synergy mechanism effectively reflect the process of digital identity construction?
RQ3: Does an individual's position within a social network serve to empower or marginalize their digital identity? Does it amplify or suppress knowledge articulation?
RQ4: Is there a misalignment between individuals' self-perceived identity and the system's role recognition? If so, how does this misalignment affect AI-HI collaboration?

## 3 Research methods

### 3.1 Research design

#### 3.1.1 Measurement methods for knowledge conversion

The entropy weight method aims to construct a weight model that describes and focuses on the distribution and uncertainty of system information. The size of the entropy value is inversely proportional to the amount of information: the higher the information content of an indicator, the greater its weight. This method is primarily used for objectively analyzing internal system changes and the relationships between indicators (Zhu et al., 2020).

The entropy weight method is widely applied in the field of management to provide auxiliary weight coefficients for decision-making, thereby improving the scientific and objective nature of decisions (Chen, 2021). Additionally, this method can be applied to management and optimization across various domains (Zhu et al., 2020). Its advantages lie in reducing human interference, enhancing applicability, efficiency, and predictability.

Weight data are typically obtained using methods such as Analytic Hierarchy Process (AHP) and Principal Component Analysis (PCA). However, AHP is subject to strong subjectivity, inconsistent ratings, and varying applicability, while PCA is primarily used for variable selection and may not accurately reflect the relative weights of variables. This study innovatively uses the entropy weight method to construct a cognitive level quantification model for collaborative innovation, with the following advantages:

##### 3.1.1.1 Objective weight assignment

Based on the principle of information entropy, this method avoids human interference and precisely quantifies the contribution weights of different cognitive levels in knowledge transformation.

##### 3.1.1.2 System adaptation

It is suitable for the cognitive interaction relationships in AI-driven innovation and supports the analysis of the non-linear collaborative effects of variables.

However, the entropy weight method is sensitive to data quality and is limited by linear assumptions (Gray, 2011). Therefore, this study implements a three-tier optimization process:

##### 3.1.1.3 Data preprocessing

Abnormal values are removed through validity tests and correlation analysis.

##### 3.1.1.4 Normalization adjustment

Data are standardized during the collection process and non-dimensionalized during the analysis phase.

##### 3.1.1.5 Model validation

By integrating the theoretical review from Section 2.2, we confirm whether the weight distribution aligns with the disruptive impact of AI on cognition.

First, the relevant indicators are standardized (see Equation 1):


(1) $$\begin{array}{l} x_{ij}=\frac{x_{ij}-\min\left(x_{j}\right)}{\max\left(x_{j}\right)-\min\left(x_{j}\right)} \end{array}$$

Here, *x*_*ij*_ represents the *i*-th indicator of sample *j*.

Next, the entropy value for each indicator is established (see Equation 2), among others $p_{ij}=\frac{z_{ij}}{\overset{n}{\underset{j=1}{\sum }}z_{ij}}$, where *n* represents the sample size.


(2) $$\begin{array}{l} e_{j}=-\frac{1}{\ln\;\left(n\right)}\overset{n}{\underset{i=1}{\sum }}p_{ij}\ln\;\left(p_{ij}\right) \end{array}$$

Finally, the weight for each indicator is calculated (see Equation 3), where *m* represents the total number of indicators.


(3) $$\begin{array}{l} w_{j}=\frac{1-e_{j}}{\overset{m}{\underset{j=1}{\sum }}\left(1-e_{j}\right)} \end{array}$$

Based on Equations 1–3, a knowledge conversion effectiveness model (Equation 4) established on the weights of cognitive levels can be derived. In this framework, the AI-HI knowledge conversion outcome (KC) represents the effectiveness of AI-HI collaboration. K_j_ represents the acquisition effectiveness of different types of cognitive levels, b represents the intercept, and ω_*j*_ represents the weights of different cognitions.


(4) $$\begin{array}{l} KC=w_{j}\cdot \sum K_{j}+b \end{array}$$

#### 3.1.2 Constructing the digital identity recognition function from a triadic synergy perspective

This study employs mathematical modeling to represent the interactive influence among the three dimensions of triadic synergy, capturing the pathway through which individual digital identities are constructed in AI-HI collaboration. As illustrated in Figure 2, an individual's digital identity can be reflected through the effectiveness of AI-HI knowledge conversion.

To capture the sensitivity of triadic synergy variables—particularly the distribution and ranking of professional skills—we adopt the Gradient Descent (GD) algorithm to model multidimensional non-linear relationships. As a core optimization technique in machine learning, GD derives the minimal loss function across multiple non-linear variables, thereby allowing the reverse estimation of each variable's contribution within AI-HI collaboration (Mohd Selamat et al., 2020). Compared with traditional multiple regression analysis, GD offers the following advantages:

##### 3.1.2.1 High-dimensional data processing

By presetting iteration counts and learning rates, the algorithm dynamically updates parameters until partial derivatives converge to a minimum, effectively improving model prediction accuracy and robustness.

##### 3.1.2.2 Innovative variable handling

Instead of treating confounding variables—such as the debated moderating/mediating role of PP—as traditional structural equation modeling does, we process them as specific parameters, avoiding methodological disputes (cf. Zhang et al., 2019; Wang et al., 2022).

##### 3.1.2.3 Feature interpretation advantages

Leveraging machine learning capabilities, we quantitatively evaluate the relative importance of different dimensions of professional skills.

To address RQ1 and RQ2, we construct a GD function (see Equation 5) to model the impact of professional skills on knowledge conversion. Here, *J*(β) represents the loss function, β1~β8 are the parameter estimates for each indicator, and β0 represents the intercept. IL (Innovation Leadership), RB (Relationship Building), TU (Tolerance for Uncertainty), and PO (Passion and Optimism) are related to soft skills. CB (cyber behavior), DA (data analysis), and FL (foreign language) are related to hard skills.


(5) $$\begin{array}{l} J(\beta )=\frac{1}{2n}\sum_{i=1}^{n}(\beta_{0}+\beta_{1}\cdot IL_{i}+\beta_{2}\cdot RB_{i}+\beta_{3}\cdot TU_{i}+\beta_{4}\cdot PO_{1}\\ \;+{\beta_{3}\cdot CB_{1}+\beta_{6}\cdot DA_{1}+\beta_{7}\cdot FL_{i}-KC_{1})}^{2} \end{array}$$

Furthermore, we construct a centrality-based function (see Equation 6) to capture the impact of social network position on collaborative innovation effectiveness, thereby addressing RQ3.


(6) $$\begin{array}{l} NC\left(i\right)=\frac{\deg\left(i\right)}{n-1} \end{array}$$

Here, *NC* (*i*) represents the relative centrality of sample iii, and deg(*i*) represents the absolute centrality of sample i. Subsequently, the GD method is used to construct the Equation 7 for the impact of centrality on dynamic knowledge conversion, reflecting the visibility of individual identity within the platform.


(7) $$\begin{array}{l} J\left(\beta_{0},\beta_{1}\right)=\frac{1}{2n}\overset{n}{\underset{i=1}{\sum }}{\left(\beta_{0}+\beta_{1}\cdot NC_{i}-KC_{i}\right)}^{2} \end{array}$$

To further address RQ3, we construct a GD function (see Equation 8) to model the influence of centrality, hard skills, and soft skills on knowledge conversion, thereby capturing how network position amplifies or suppresses the effectiveness of professional skills.


(8) $$\begin{array}{l} J\left(\beta \right)=\frac{1}{2n}\overset{n}{\underset{i=1}{\sum }}{\left(\beta_{0}+\beta_{1}\cdot NC_{i}+\cdots +\beta_{8}\cdot FL_{i}-KC_{1}\right)}^{2} \end{array}$$

To address RQ4, we construct Equation 9 to represent the subject-object matching problem in digital identity. In this model, *PP* denotes proactive personality.


(9) $$\begin{array}{l} J\left(\beta \right)=\frac{1}{2n}\overset{n}{\underset{i=1}{\sum }}{\left(\beta_{0}+\beta_{1}\cdot NC_{i}+\cdots +\beta_{8}\cdot FL_{i}-KC_{1}\right)}^{2} \end{array}$$

Equations 5, 7–9 are loss functions concerning regression relationships, and their parameters need to be iteratively updated using the GD method (see Equation 10). Here, α is the learning rate, and $\frac{\partial_{J\left(\beta \right)}}{\partial_{\beta }}$ is the partial derivative of the loss function with respect to the regression coefficients.


(10) $$\begin{array}{l} \beta :=\beta -\alpha \frac{\partial_{J\left(\beta \right)}}{\partial_{\beta }} \end{array}$$

To address the algorithm's limitations, we implement a three-tier optimization strategy:

##### 3.1.2.4 Sensitivity to initial values

Running the model algorithm at least 100 times to compute the standard deviation and mean of coefficients.

##### 3.1.2.5 Overfitting control

Employing the *Early Stopping* method to prevent overfitting by testing different parameter settings.

##### 3.1.2.6 Parameter tuning

Utilizing Goodfellow's (2016) dynamic adjustment framework to balance model convergence speed and training efficiency.

In summary, the constructed models are closely aligned with the research questions, with the weights of each dimension reflecting the identification of individual roles in AI-HI collaboration. Variations in these weights indicate changes in digital identity construction under the influence of confounding variables.

#### 3.1.3 Personalized digital identity matrix based on residual-macthing analysis

To further address research questions RQ1 through RQ4, we first identify AI-resilient skills using Equation 5 through Equation 10. We then examine how the interaction among individual motivation, structural position, and resilient skills leads to inconsistencies between system-assigned roles and self-perceived identities. This divergence is analyzed in terms of its impact on AI-HI role allocation.

Based on this premise, we construct an identity recognition framework grounded in residual analysis and matching calculation. The specific steps are as follows:

First, we establish the residuals of knowledge innovation effectiveness (see Equation 11).


(11) $$\begin{array}{l} \text{Residual}=KC_{\text{actual}}-KC_{\text{pred}} \end{array}$$

Among them, *KC*_actual_ represents actual effectiveness (related to Equation 4), *KC*_pred_ representing predictive effectiveness (Equation 12), ∑β_*n*_· Skills _*n*_ represents key skills.


(12) $$\begin{array}{l} KC_{\text{pred}}=0.04NC+0.05PP+\sum \beta_{n}\cdot {\text{Skills}}_{n} \end{array}$$

Then, we used PP as a moderator to build the NC-Skills-PP match calculation (Equation 13):


(13) $$\begin{array}{l} \text{ Mathscore}=\\ \frac{\left(NC\times \sum \beta_{n}\cdot {\text{Skills}}_{n}\right)\times \left(1+\alpha \times PP\right)-\min\left(\text{Matchscore}\right)}{\max\left(\text{Matchscore}\right)-\min\left(\text{Matchscore}\right)}\times 2 \end{array}$$

By applying GD, we integrate residual analysis and matching calculation into the theoretical framework of digital identity construction. The difference between predicted and actual identity expression *KC*_*actual*_ termed identity matching deviation. Residual serves as the basis for subsequent classification in the personalized collaboration matrix.

Based on the combination of residuals and matching scores, each sample is categorized into one of three types, as shown in Table 1.

**Table 1:** Interpretation of digital identity profiles based on the combination of residuals and matching.

**Category**	**Classification logic**	**Theoretical interpretation**
Low performers	High residual + Low matching	Indicates that both the system and the individual fail to recognize the role, leading to weak identity activation or alignment
Peripheral experts	High matching + High residual	Possess relevant skills but hold a marginal structural position; identity expression is misaligned with system recognition
Core innovators	High matching + Low residual	Demonstrates high triadic alignment, with strong knowledge articulation and consistent platform-based identity recognition

### 3.2 Explanation of variables

#### 3.2.1 Network centrality, proactive personality and professional skill

This study employs nomination surveys and interviews to collect data on NC. The scale for NC is based on the work of Soda and Zaheer (2012), using the nomination method and presenting the data in matrix form, as referenced in the study by Cangialosi et al. (2021).

The measurement of PP is based on the scale designed by Seibert et al. (2001). According to previous experimental records, the consistency coefficient between the items of the scale is 0.89. This study conducted a convergence test on the scale design, selecting 8 questions with a 6-point attitude scale.

Cyber Behavior (CB), Data Analysis (DA), and Foreign Languages (FL) represent the critical impact of hard skills on innovation. Based on Hendarman and Cantner (2018), this study collects measurable data to represent hard skills. The measurement of soft skills follows the scale designed by Hendarman and Cantner (2018) and gathers data through questionnaires. Item 1 measures Innovation Leadership (IL), Item 2 measures Relationship Building (RB), Item 3 measures Tolerance for Uncertainty (TU), and Item 4 measures Passion and Optimism (PO). While referencing Hendarman's scale, this study makes adjustments, setting the attitude scale at 6 levels. A detailed structure of the questionnaire design can be found in Supplementary Table S1.

#### 3.2.2 Quantification of cognition in knowledge conversion

As summarized earlier, knowledge conversion is a significant manifestation of knowledge innovation. Therefore, this study employs the After Action Review (AAR) method to conduct a feedback-based evaluation of the knowledge conversion process. The method is applied with reference to Keiser and Arthur (2021), combining Bloom's cognitive model to perform AAR analysis and extract results from the knowledge conversion process. For detailed reference, see Supplementary Table S2.

### 3.3 Survey participants

The experiment of this study was conducted between 2022 and 2024, targeting companies and employees within the Xiaoguishan Financial Industry Park in Wuhan. The enterprises in this industrial park predominantly belong to symbiotic industries, including project consulting, digital technology, finance, and cultural investment. To align with the digital transformation of industries, the park's enterprises engaged in digital transformation-related work during the pandemic period. The survey involved a total of 455 participants, with 418 valid samples collected. These participants were distributed across 7 community networks, as detailed in Table 2.

**Table 2:** Demographics of the survey respondents.

**Variables**	**AVG**	**S.D**	**Industrial distribution**	**Percentage**
Gender	1.67	0.47	Financial	25%
Age	2.06	0.32	Cultural tourism	25%
Education (Edu.)	1.90	0.41	Digitization	13%
Job description (JD)	1.72	1.35	Project consultancy	17%
Forward transfer (FT)	1.00	0.05	Others	20%

N = 418. Gender: 1 = male, 2 = female. Age (years old): 2 = 19–29, 3 = 30–39. Education: 1 = less than bachelor's degree, 2 = bachelor's degree. Job Description: 1 = General Employee, 2 = Departmental Management. Forward Transfer: 1 = had relevant experience and knowledge base.

It should be noted that most of the participants are interns from Wuhan University of Business and Technology, who underwent similar training and cognitive internships prior to their placement (the average value of forward transfer is 1.00, indicating the coverage of the preliminary training). The majority of participants volunteered to take part, and our survey activities were governed by the regulations of the Hubei Provincial Department of Education project (2021GA078).

## 4 Data analysis

### 4.1 Data preprocessing

#### 4.1.1 Reliability and validity testing

Table 3 includes the KMO values, total variance explained, AVE, and CR values for the variables in the hypothetical research model. The KMO values indicate a strong correlation among the indicators within the four latent variables of the hypothetical model. The total variance explained (single component) is above 50%, demonstrating that the indicators sufficiently explain the latent variables. AVE values are all above 0.5, and CR values exceed 0.6, indicating that the convergent validity meets the requirements, further validating the scientific rigor of the selected indicators.

**Table 3:** Reliability and validity of latent variables.

**Variables**	**Items**	**KMO**	**TVE**	**AVE**	**CR**
Knowledge conversion (KC)	6	0.83	77.37 %	0.77	0.93
Proactive personality (PP)	8	0.89	53.70 %	0.53	0.90
Soft skill (SS)	4	0.77	63.00%	0.63	0.87
Hard skill (HS)	3	0.64	65.20 %	0.65	0.85

The analysis results are derived from SPSS and EXCEL. KMO > 0.6; TVE (%) > 50; AVE > 0.5; CR > 0.6.

#### 4.1.2 Correlation analysis

**Figure 3:**
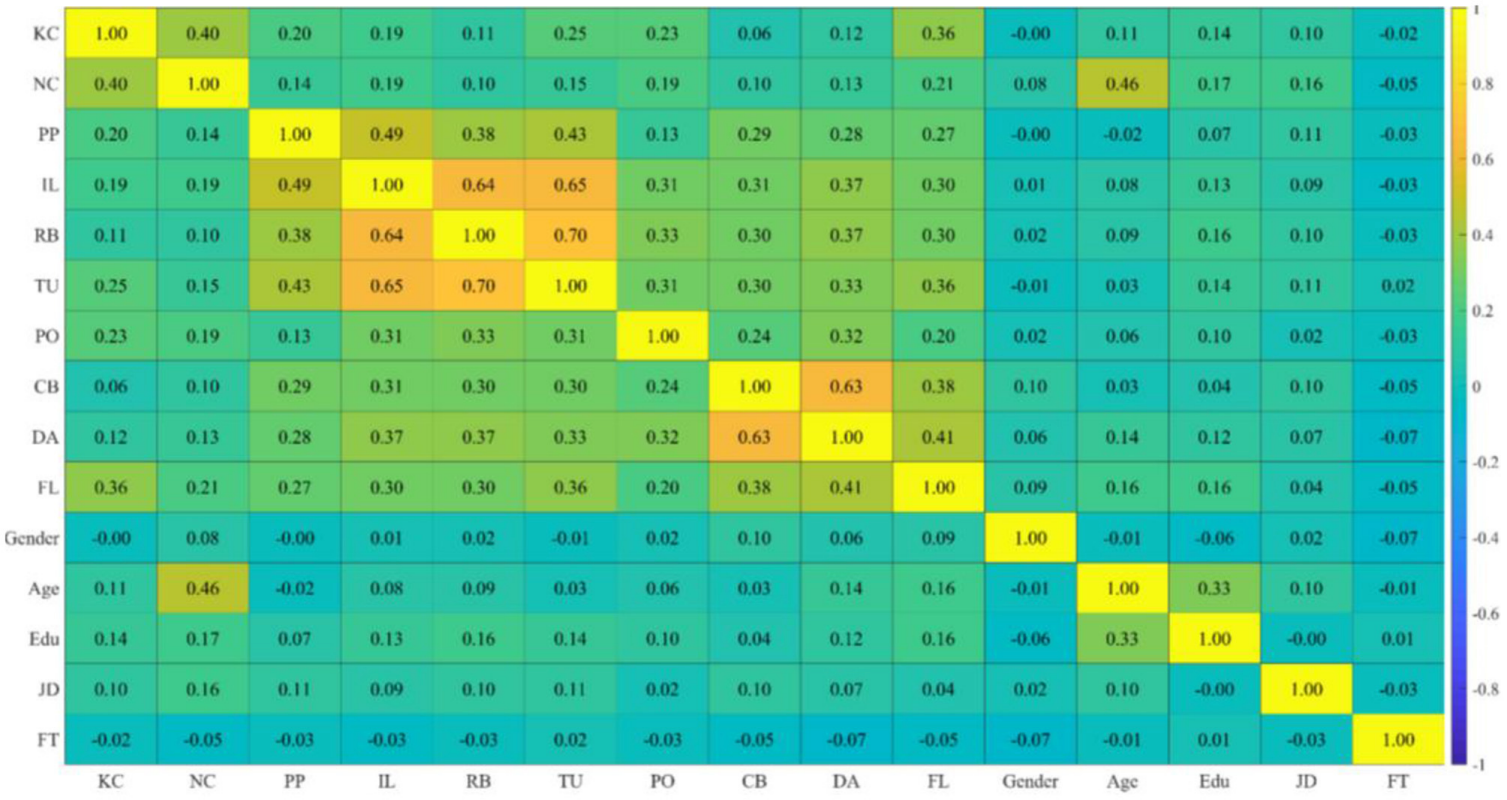
Heat map of correlation. The color gradient represents the strength of Pearson correlation (ranging from −1 to +1, from blue to yellow). For variable abbreviations, see Tables 2, 4; NC denotes network centrality, and PP refers to proactive personality.

Based on the heat map of variable correlations (see Figure 3), it is evident that all variables, except for demographic variables, exhibit a certain degree of positive correlation. According to Gelman and Hill (2006, pp. 20–21), the entropy weight method and GD can eliminate the influence of demographic variables on the hypothetical research.

### 4.2 Quantitative cognitive analysis in the AI-HI collaboration process

**Figure 4:**
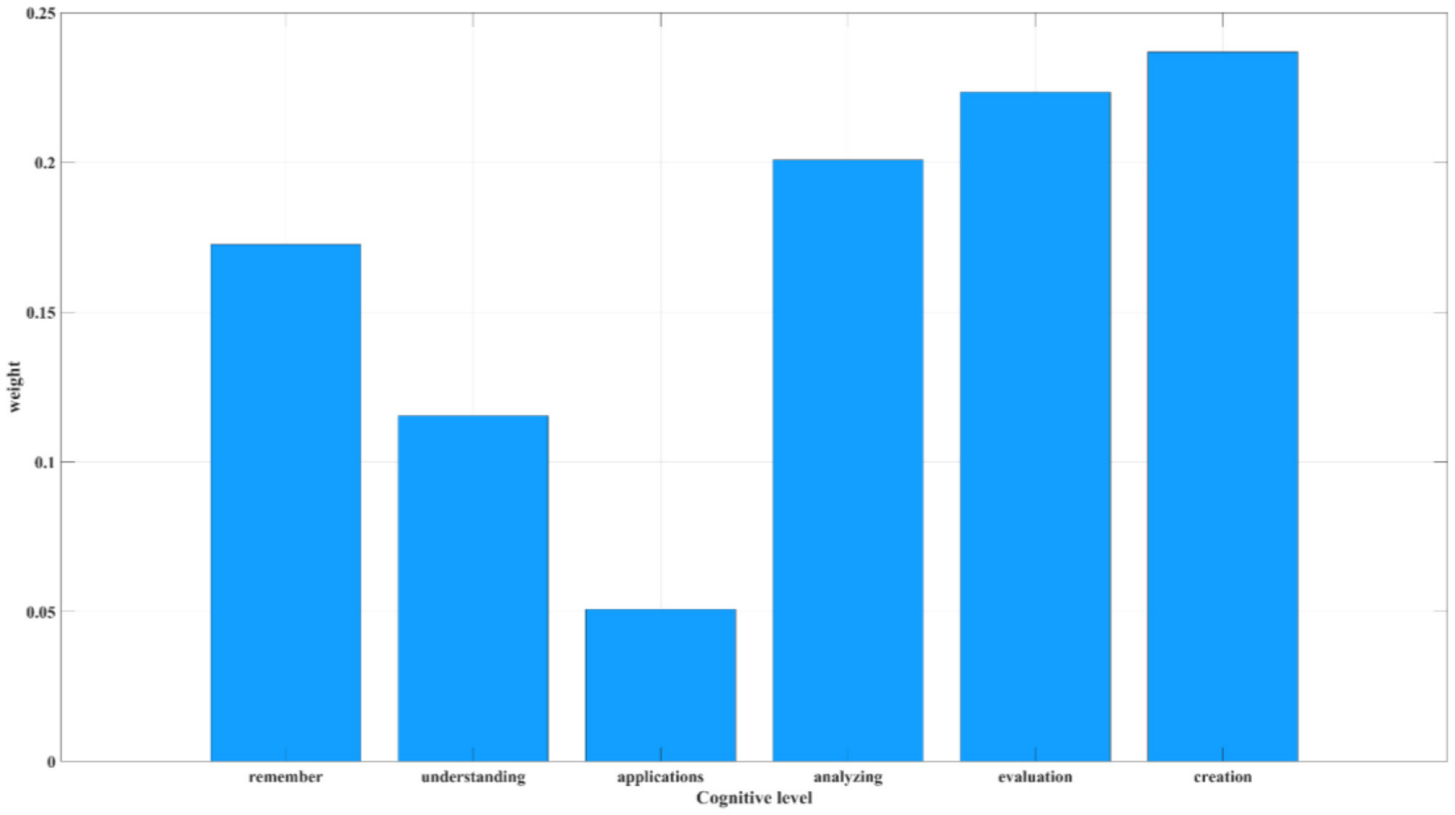
Weighted contributions of individual cognitive categories in AI-HI collaboration (Based on entropy weight method). The six cognitive levels are classified according to Bloom's taxonomy. The weight values represent each level's contribution to AI-HI knowledge conversion. Higher-weighted levels—creation, evaluation, and analysis—correspond to higher-order cognition. These results indicate that AI-HI collaboration relies more heavily on complex cognitive processes.

Based on Equation 1 through Equation 3, the entropy weighting method was used to quantify individual contributions at different cognitive levels within AI-HI collaboration (see Figure 4). The analysis revealed the following weighted contributions: creation (0.2369) > evaluation (0.2235) > analysis (0.2008) > remembering (0.1726) > understanding (0.1154) > applying (0.0508). These results indicate that, compared to lower-order cognitive processes (remembering, understanding, and applying), higher-order cognition—represented by creation, evaluation, and analysis—plays a more significant role in AI-HI collaborative innovation. This finding reflects AI's substitution effect on lower-order cognition and its enhancement of higher-order cognitive functions (Hu et al., 2021; Asrifan et al., 2024; Jia et al., 2024; Ritala et al., 2024).

Overall, this validates the model's rationality in quantifying AI-HI collaboration effectiveness. Finally, according to Equation 4, the weighted values across cognitive levels can be integrated to predict the AI-HI collaborative innovation effectiveness, represented by the KC value.

### 4.3 Gradient regression analysis results

Based on responses to RQ1 and RQ2, this study employed GD to model the relationship between professional skills and collaborative innovation effectiveness (Equation 5). The model was iterated 100 times to reduce sensitivity to initial values and improve prediction stability. The results are presented in Table 4.

**Table 4:** Contributions of professional skills in the AI-HI collaboration process.

**Variable**	**Definition**	**Coefficient**	**Standard error**	**T value**	**P value**
IL	Innovation leadership	0.02	0.01	1.54	0.12
RB	Relationship building	−0.03^*^	0.01	−2.19	0.03
TU	Tolerance for uncertainty	0.04^**^	0.01	3.14	0.00
PO	Passion and optimism	0.03^**^	0.01	4.63	0.00
CB	Cyber behavior	−0.02	0.01	−1.80	0.07
DA	Data analysis	0.00	0.01	0.39	0.70
FL	Foreign language	0.07^**^	0.01	7.32	0.00

The analysis results are derived from MATLAB. A smaller standard error indicates a more precise estimate of the regression coefficients; the T value is used to test whether the regression coefficients are significantly different from zero;

** *p* < 0.01,

* *p* < 0.05.

Regarding skill distribution, among soft skills, IL, TU, and PO, along with the hard skill of FL, showed significant positive effects on AI-HI knowledge conversion effectiveness (KC). Notably, PO (β = 0.03^**^) and FL (β = 0.07^**^) stood out prominently.

These findings reveal: (1) FL plays a critical role in overcoming knowledge barriers and facilitating knowledge flow, serving as a key factor in AI-HI cross-boundary collaboration; (2) PO functions as a stabilizing mechanism for individual identity, significantly enhancing individuals' capacity to engage with AI-HI collaborative content.

These results support RQ1 by identifying PO and FL as the most AI-resistant professional skills, capable of consistently expressing individual identity and knowledge value.

Further, by integrating Equations 6–9, this study examined the effects of structural position (NC) as a dimension of digital identity recognition, and individual intrinsic motivation (PP) on AI-HI collaborative innovation effectiveness. The findings are as follows:

1. The effect of NC on KC was 0.07^**^, indicating that structural position characteristics positively contribute to AI-HI collaboration. This result supports the first part of RQ3, confirming that social network position has an empowering effect on digital identity recognition.
2. Under the influence of NC (detailed results of the regression coefficients are reported in Supplementary Table S7), TU (0.03^**^) and FL (0.05^**^) showed significant positive impacts on KC. This suggests that, with structural position empowerment, individuals' knowledge articulation is further strengthened, addressing RQ2 regarding the constructive role of the triadic synergy in digital identity formation.
3. Within the triadic synergy framework, regression coefficients for PP, NC, FL, and TU were 0.05^**^, 0.04^**^, 0.05^**^, and 0.03^*^ respectively (detailed results of regression coefficients are shown in Supplementary Table S8). This finding responds to the combined research questions RQ2 and RQ3, illustrating the logic by which the triadic synergy influences individual digital identity recognition.

**Note:** Details regarding model parameter settings, gradient optimization, and overfitting tests are provided in the Supplementary Tables S3–S6 and Supplementary Figures S1–S4. These materials are intended to validate the model's stability and do not affect the presentation of the main data analysis results.

### 4.4 Parameter settings for confounding variables and skill priority optimization

To further strengthen the explanatory power of structural position and individual intrinsic motivation on knowledge articulation, this study employed a fixed confounding variable parameter combined with gradient-based re-optimization to reassess the contribution of professional skills. Specifically, the parameters for NC (0.04^**^) and PP (0.05^**^) were fixed. Using gradient regression, the triadic synergy model in Equation 9 was iterated 500 times.

**Figure 5:**
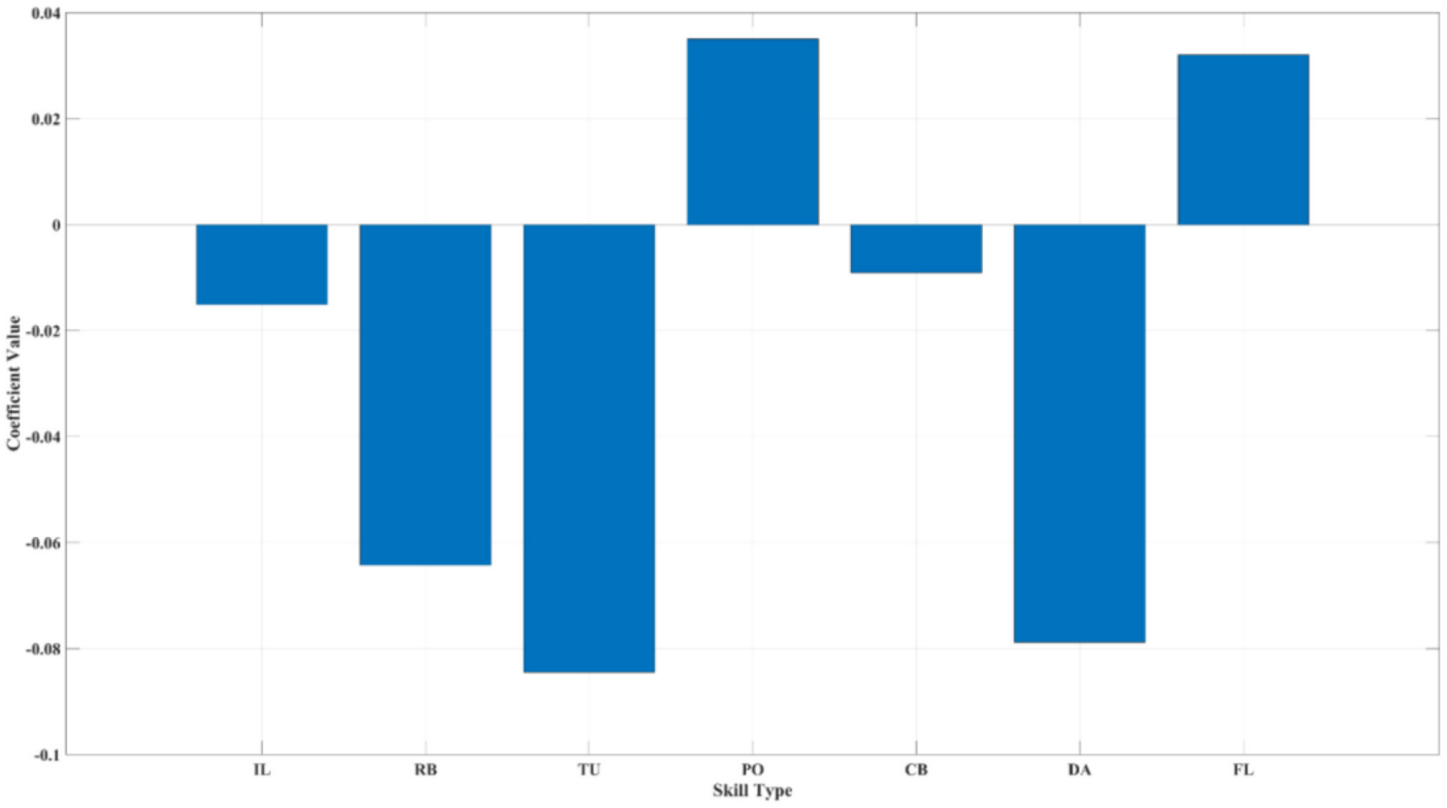
Relative contribution ranking of professional skills based on gradient optimization (500 iterations). The Y-axis represents the gradient descent regression coefficients. Both *FL* and *PO* retain significant advantages even under the control of confounding variables *NC* and *PP*, indicating that they are AI-resistant skills. Detailed results of regression coefficients are shown in Supplementary Table S9.

The analysis results (see Figure 5) indicate that, under the conditions of PP and NC, PO (0.04^**^) and FL (0.03^**^) emerge as the most “AI-resistant” skills in AI-HI collaboration. This finding further deepens the response to RQ1, demonstrating that “AI-resistant” skills continue to have a stable driving effect on AI-HI collaborative innovation effectiveness even when controlling for structural position and individual motivation. This reflects that truly AI-resistant skills possess strong digital identity expression.

### 4.5 Decision strategy matrix: classification based on digital identity recognition

**Figure 6:**
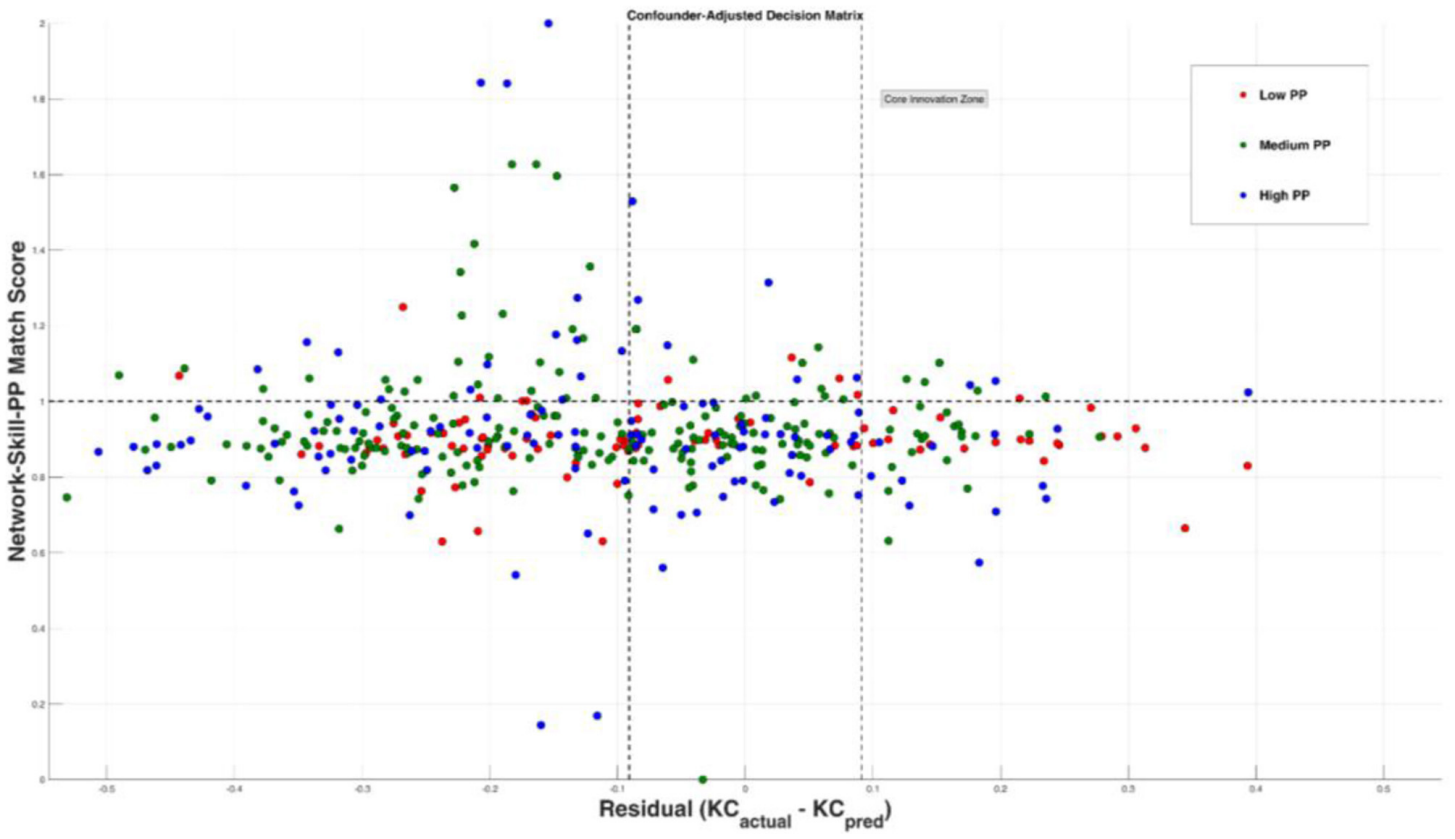
Sample size distribution based on residuals and matching degree. The X-axis represents the degree of identity matching, while the Y-axis indicates the residuals of innovation effectiveness. Marginal experts are primarily located in the “high residual + medium matching” quadrant, revealing a structural misalignment in identity recognition.

In response to RQ4, this study constructs a personal digital identity recognition matrix by combining residuals and matching degrees. The recognition matrix categorizes roles within AI-HI collaboration. Specifically, through parameter settings of confounding variables and skill priority optimization, PO (0.04^**^) and FL (0.03^**^) are identified as important indicators of individual knowledge articulation. Then, based on Equations 11–13, the distribution of sample sizes, strategy classifications, and adaptation recommendations for digital identity recognition are obtained (see Figure 6, Table 5).

**Table 5:** Digital identity strategy types and adaptation recommendations.

**Strategy type**	**Low PP group (15 %)**	**Mid-PP group (45%)**	**High PP group (40 %)**
Core innovators	0	0.01	0.99
Marginal experts	0.12	0.23	0.65
Low-efficiency individuals	0.82	0.15	0.03
**Residuals**\**Match**	**Low match (MatchScore**<**0.8)**	**Medium match (0.8** ≤ **MatchScore** ≤ **1.2)**	**High match (MatchScore** > **1.2)**
High residual (Residual > +0.3)	Marginal experts: AI-enhanced breakthroughs	Core innovators: strategic ownership	Core innovators: strategic ownership
Medium residual (−0.3 ≤ Residual ≤ +0.3)	Routine optimisation	Progressivity	Structured tasks
Low residuals (Residual < −0.3)	Low-efficiency individuals: redeployment/elimination	Standardized training	Rotation activation

Table 5 details the proportions and response strategies for Core Innovators, Marginal Experts, and Low Performers. These results demonstrate that digital identities under the triadic synergy framework can be quantitatively identified and dynamically adjusted, providing effective recommendations for role allocation in AI-HI collaboration.

## 5 Discussion, conclusion, and outlook

### 5.1 Summary of key findings

This study investigates digital identity recognition under AI-HI collaborative innovation by constructing a triadic mechanism involving knowledge articulation, structural position, and personal motivation. Compared to previous research, it offers key findings and extensions in the following aspects:

First, we redefine “AI-resistant” professional skills, breaking through the traditional binary perspective of soft vs. hard skills. Prior studies primarily focused on the structural differences between soft and hard skills (Hendarman and Cantner, 2018; Alekseeva et al., 2021; Fletcher and Thornton, 2023). Our model, incorporating confounding variables, reveals that FL and PO exhibit more stability in AI-HI collaboration. This finding acknowledges the unique roles of both hard and soft skills in AI-HI collaboration and highlights that highly expressive and system-recognized skills are critical in constructing digital identity. Furthermore, while Alekseeva et al. emphasize the key roles of DA and CB in the digital economy, our findings suggest these skills are increasingly supplanted by AI, whereas FL and PO show stronger resistance to replacement. This provides empirical support for research on AI-HI complementarity.

It is important to clarify that FL as an AI-resistant skill may be easily misunderstood—after all, LLMs are widely recognized as one of AI's core strengths. However, FL in this study is not limited to linguistic proficiency *per se*. Instead, it represents a deeper capability: the power to integrate knowledge across boundaries (Nonaka and Yamaguchi, 2022). In contexts where AI plays a dominant role but lacks contextual adaptability and cultural discernment, FL is more likely to function as a technological tool for identity expression and bridging, enabling individuals to serve as connectors in human-AI collaboration.

Second, we propose a collaborative construction logic of digital identity, critiquing prior studies' reliance on “system-label” identity assignment. Typically, role recognition and assignment in human–AI collaboration are attributed to system-level configurations (e.g., Alowais et al., 2023; Bharatha et al., 2024). However, such studies often overlook the interactive dynamics among self-driven motivation, collective dynamics, and cognitive performance. Our study argues that individual digital identity emerges from the synergy of internal motivation, structural position, and knowledge articulation. Moreover, by combining residual and matching degree analyses, we concretize the discrepancy between system recognition and individual identity, empirically challenging the simplistic “system-label” model.

Third, this study reveals a bidirectional “empowering/inhibiting” effect of structural position on individual knowledge articulation, offering a contextualized extension of the “structural empowerment logic” within existing social capital theory. In contrast to prior studies (e.g., Cangialosi et al., 2021), our findings suggest that in hybrid human–AI social networks, NC does not uniformly activate knowledge articulation. Its amplifying effect depends on skill fit and personal motivation. The identification of “marginal experts” in Figure 6 and Table 5 illustrates that, even individuals with high-level skills may find their identity expression suppressed—particularly when they occupy structurally marginal positions and lack proactive personal drivers.

Additionally, RB also contributes to the expression of structural position. However, in our model, it exhibits a negative effect (β = −0.03). We speculate that in highly collaborative and digitized task environments, an overreliance on RB may lead to reduced communication efficiency, blurred role boundaries, and a tendency to avoid conflict. This finding aligns with previous research suggesting that unstructured social interactions in virtual teams can undermine collaborative performance (Morrison-Smith and Ruiz, 2020; Caldwell et al., 2022).

Fourth, establishing an intervention mechanism for digital identity recognition addresses the current limitations in quantifying AI–HI collaborative innovation effectiveness and the challenges posed by the “black box” nature of evaluations. Existing studies largely rely on self-reported questionnaires to measure collaborative innovation outcomes (Chowdhury et al., 2022; Pham et al., 2024), which do not fully capture the cognitive dynamics and human–AI role distinctions inherent in AI–HI collaboration. This study employs the entropy weight method to develop a more objective evaluation framework for human–AI collaborative effectiveness. Furthermore, through a residual–matching analysis matrix, it identifies three distinct groups—core innovators, marginal experts, and low performers. This approach not only enhances the objectivity and explanatory power of AI-driven performance assessments but also offers practical insights for the design of AI–HI symbiotic mechanisms and human–AI role allocation.

### 5.2 Theoretical contributions

First, this study reconstructs the AI-HI collaboration theoretical framework from the perspective of digital identity. It breaks the traditional binary paradigm of technological determinism and capability determinism, proposing a triadic synergy model of “Motivation-Structure- Articulation,” embedding digital identity construction into the AI-HI symbiotic ecosystem.

Second, methodological innovations are made by integrating entropy weighting, gradient descent, and residual-matching techniques. This not only improves self-reported measurement approaches but also innovatively quantifies digital identity recognition in AI-HI collaborative scenarios.

Finally, the study redefines the skill substitution effect. It argues that language proficiency and emotional intrinsic motivation serve as more robust AI-resistant skills in AI-HI collaboration than procedural skills such as data analysis. This shift helps advance the research paradigm from “technical adaptation” toward “identity expression” in professional skills studies.

### 5.3 Management insight

First, based on the residual-matching decision matrix, a personalized talent identification and allocation mechanism can be constructed to enable precise interventions for “core innovators,” “marginal experts,” and “low performers.” Core innovators should be granted greater autonomy; low performers should undergo standardized training, job rotation, or optimization; and marginal experts should be supported through a structured mentorship system.

Second, in designing AI-driven collaborative effectiveness, structural adjustments of AI-HI social networks are necessary, especially by providing platforms and collaboration opportunities for high-skill but low-position marginal experts. To enhance the structural recognition of marginal experts, cross-departmental task forces should be established to increase their involvement in key roles. Additionally, network-building initiatives can be implemented to facilitate their integration into core collaboration circles.

Finally, combining residual-matching strategies supports sustainable talent-resource allocation. In AI-HI skill training, priority should be given to cultivating AI-resistant skills

### 5.4 Shortcomings and outlook

First, the assessment of soft skills remains subjective. The evaluation methods and criteria for soft skills in this study are primarily based on questionnaires, which are evidently overly subjective. Therefore, future research should adopt more objective approaches to quantify soft skills, such as technology-driven soft skill assessments (Altomari et al., 2023).

Secondly, the triadic synergy model effectively explains digital identity construction in AI-HI collaboration. However, variables such as AI model performance and task scenarios require further refinement. Moreover, this study primarily employs quantitative analysis and lacks the integration of qualitative data (e.g., interviews), which is essential for enhancing the explanatory power of the model. At the same time, future research should consider conducting group-based comparative tests on the residual–matching approach to improve the model's robustness and rigor.

Third, the cross-sectional nature of this study limits a comprehensive understanding of AI technology development and iteration over time. Moreover, the impact of Explainable Artificial Intelligence (XAI) on knowledge innovation exhibits distinct phase-specific characteristics (Mancuso et al., 2025). Therefore, future research should incorporate longitudinal studies to observe temporal changes in relevant variables.

Fourth, this study did not quantitatively assess the technical characteristics of AI systems, although existing research highlights their close relationship with digital identity construction and task adaptation (Shneiderman, 2022; Huang and Rust, 2024). The diversity of AI-HI collaborative systems poses a significant challenge for current research. Future studies should aim to develop appropriate and generalizable methods to quantify AI system features, enabling a more comprehensive characterization of AI-HI collaboration mechanisms.

Finally, while this study's sample size is substantial, it is limited by the convenience of data collection, resulting in a relatively homogeneous respondent background and role distribution. Although participants generally received relevant training, many occupied peripheral positions within social network structures and had insufficient understanding of work processes. These factors inevitably impact the knowledge expression system and collaboration effectiveness. Future research should consider cross-validating findings across multiple industries and among formal employees to further strengthen the robustness and generalizability of the conclusions.

## Data Availability

The original contributions presented in the study are included in the article/Supplementary material, further inquiries can be directed to the corresponding author.
