# Supplementary material for: Reframing individual roles in collaboration: digital identity construction and adaptive mechanisms for resistance-based professional skills in AI-human intelligence symbiosis
Source: Front Psychol. 2025 Aug 8;16:1652130. doi: 10.3389/fpsyg.2025.1652130 (PMC12372334; doi:10.3389/fpsyg.2025.1652130)
Supplement: Supplementary file 1 [file Data_Sheet_1.pdf]

Supplementary Material

Appendix A Variable Definitions and Assessments

Table S1. Interpretation of independent variables.

Variables	Items	Refer
Network centrality	1. Who do I usually ask for advice when I encounter difficulties in the course of my work and study?<br>2. Who do I usually discuss relevant issues with when dealing with daily business?<br>3. Who gives me advice when I encounter difficulties?	Soda and Zaheer (2012); Cangialosi et al.(2021)
Proactive personality	1. I am always exploring new ways to improve my life.<br>2. I am a powerful force for constructive change wherever I am.<br>3. There are few things more exciting than seeing my ideas come to life.<br>4. If I see something I don’t like, I’ll do my best to change it.	Seibert et al.(2001)

	5. If I believe in something strongly, no matter what the chances of success are, I'll do my best to change it.<br>6. I will stick to my idea even if others are against it.<br>7. I am good at recognizing opportunities.<br>8. If I strongly believe in an idea, no obstacle can stop me	
Hard skills	1. Cyber Behavior (CB), referenced by NCRE[1]-2 scores as a reference.<br>2. Data Analysis (DA), referenced by the score of the practical computer test.<br>3. Foreign Languages (FL), using CET[2]-4 scores as a reference.	Hendarman and Cantner(2018)
Soft Skills	1. Innovation Leadership (IL), I have influenced innovation at my workplace.<br>2. Relationship Building (RB), I have made extra efforts to develop business and professional relationships with partners both inside and outside the company.	

[1]NCRE: National Computer Rank Examination

[2]CET: College English Test

	3. Tolerance for Uncertainty (TU), I feel comfortable making decisions in uncertain situations.<br><br>4. Passion and Optimism (PO), Even when working in a team that is not collaborative, I can make forward progress.

Table S2. AAR assessment of knowledge conversion.

Cognitive level	Test Design	Reference	Criteria
Remembering	1.List commonly used models and equations	listing	Based on remembering what was learned
	2. Questionnaire Design Structure and Notes	Retrieving/ Identifying	
Understanding	1. List at least 3 ways to survey data and describe the scope of application	Summarising/ Inferring	Depending on the understanding of the different approaches
	2. Compare the advantages and disadvantages of different analytical methods, their corresponding data types and usage Scenarios	Comparing/ Explaining	
	3. Listing of tools and websites on social networking, knowledge organization and data acquisition	Social networking/ Boolean Searching/ Categorising/ Subscribing	Proper selection and application of learning tools and components
Applications	1. Demonstrate operations: how did you find the data?	Using/implementing/showing	Rate ability to uncover and analyze data through

	What channels or tools were used to find the data<br><br>2.Demonstrate operations: data processing; app or tool		presentations and demonstrations
Analyzing	1.Data discovery and corresponding analytical methods are described, and a set of programs is recommended and justified	Organising/Deconstructing	Rating based on match of scenarios, data types, analytics and insights
Evaluation	1.Which partners gave me which suggestions for this conclusion? How did I interpret these suggestions (self-reflection)?	Collaborating and networking	Rating of judgment and reflection based on comments on others' contributions
Creation	1.Report on the practice	Directing and producing	Rate the report for predictive, practical, instructive and creative

Source: Authors' own creation

Appendix B Parameter Tuning and Validation

Table S3. Parameter tuning for Eq. (5).

**Items**	**Parameter setting**	**Test1**	**Test2**	**Test3**	**Test4**
Learning rate	0.01	0.1	0.001	0.0001	0.01
Iteration	1000	500	3000	5000	2000
MSE	0.0344	NaN	0.0357	0.0389	0.0336
Mean regression coefficient for soft skills	0.0148	NaN	0.0176	0.0198	0.0126
Mean regression coefficient for hard skills	0.0201	NaN	0.0217	0.0183	0.0177

Source: Authors' own creation

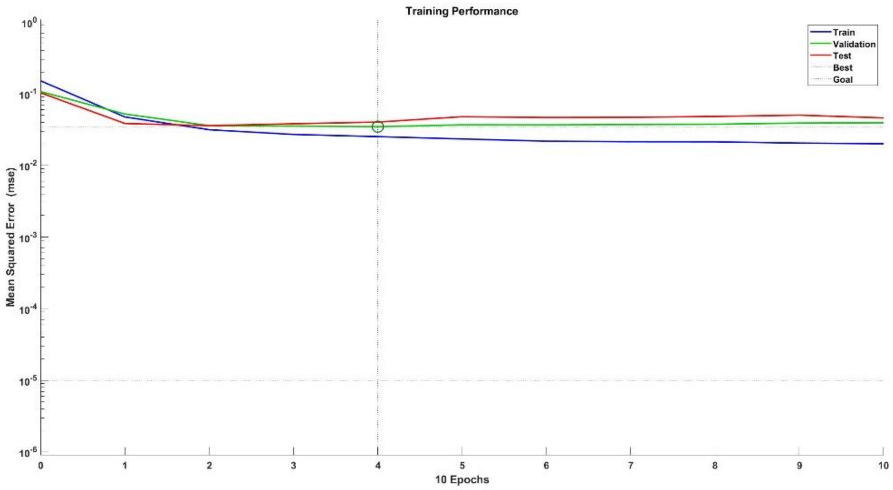

Figure S1. Eq. (5) overfitting validation (learning rate 0.01, 6000 iterations).

Source: Authors' own creation

Table S4. Parameter tuning for Eq. (7).

Items	Parameter setting	Test1	Test2	Test3	Test4
Learning rate	0.01	0.1	0.001	0.0001	0.01
Iteration	1500	500	3000	5000	2000
MSE	0.033705	0.033688	0.039819	0.054458	0.033689
Regression coefficient	0.07	0.06	0.11	0.15	0.06

Source: Authors' own creation

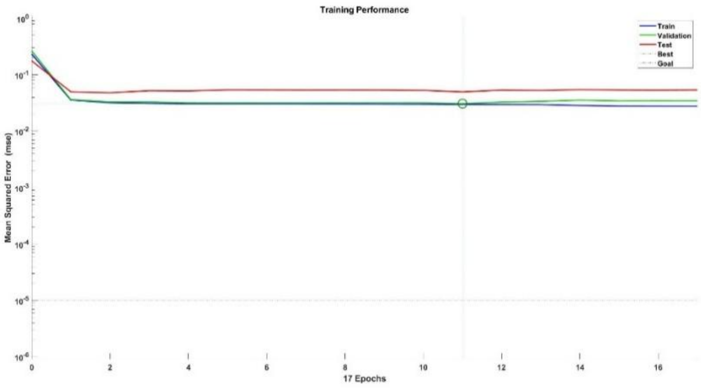

Figure S2. Eq. (7) overfitting validation (learning rate 0.01, 5000 iterations).

Source: Authors' own creation

Table S5. Parameter tuning for Eq. (8).

Items	Parameter setting	Test1	Test2	Test3	Test4
Learning rate	0.01	0.1	0.001	0.0001	0.01
Iteration	2000	500	3000	5000	1000
MSE	0.029113	NaN	0.031996	0.034528	0.030224

Source: Authors' own creation

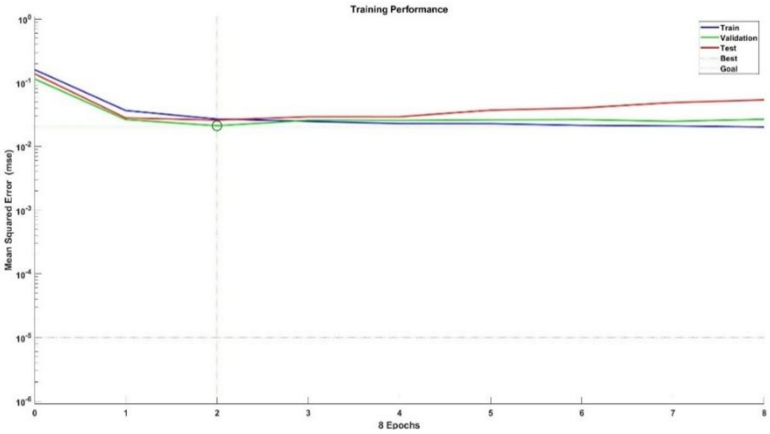

Figure S3. Eq. (8) overfitting validation (learning rate 0.01, 6000 iterations).

Source: Authors' own creation

Table S6. Parameter tuning for Eq. (9).

Items	Parameter setting	Test1	Test2	Test3	Test4
Learning rate	0.01	0.1	0.001	0.0001	0.01
Iteration	2000	500	3000	5000	1000
MSE	0.028225	NaN	0.029127	0.031979	0.28606

Source: Authors' own creation

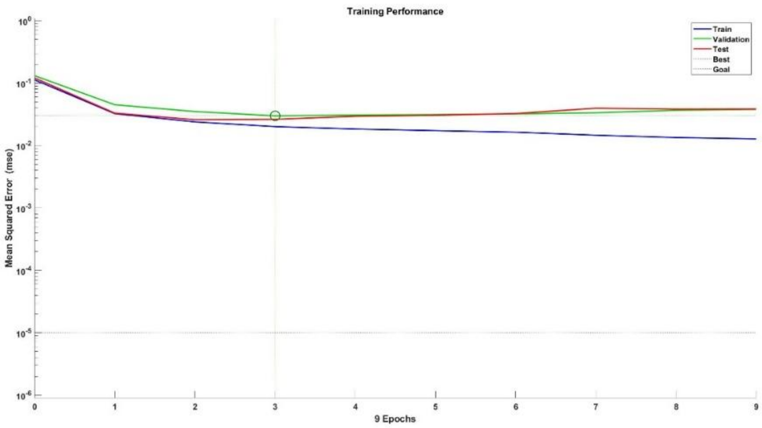

Figure S4. Eq. (9). overfitting validation (learning rate 0.01, 6000 iterations).

Source: Authors' own creation

Appendix C: Index weight

Table S7. Regression coefficients based on Eq. (8) (learning rate of 0.01, number of iterations 2000, tested 100 times).

Variable	Definition	Coefficient	Standard Error	T Value	P Value
Intercept		0.2	0.0422	4.6890	0.0000
NC	Network Centrality	0.05	0.0070	6.7930	0.0000
IL	Innovation Leadership	0.01	0.0102	0.9840	0.3258
RB	Relationship Building	-0.02	0.0121	-1.7453	0.0819
TU	Tolerance for Uncertainty	0.03	0.0119	2.4508	0.0148
PO	Passion and Optimism	0.03	0.0084	1.3056	0.1926
CB	Cyber Behaviour	-0.01	0.0091	-1.4474	0.1487
DA	Data Analysis	0.00	0.0108	0.5688	0.5699
FL	Foreign Language	0.06	0.0096	4.8443	0.0000

Source: Authors' own creation

Table S8. Regression coefficients based on Eq. (9) (learning rate of 0.01, number of iterations 2000, tested 100 times).

Variable	Definition	Coefficient	Standard Error	T Value	P Value
PP	Proactive Personality	0.05	0.0131	4.1676	0.0000
NC	Network Centrality	0.04	0.0069	6.3860	0.0000
IL	Innovation Leadership	-0.04	0.0104	-0.1871	0.8517
RB	Relationship Building	-0.03	0.0119	-1.9919	0.0472

TU	Tolerance for Uncertainty	0.03	0.0118	1.9723	0.0494
PO	Passion and Optimism	0.02	0.0083	1.1626	0.2458
CB	Cyber Behaviour	-0.02	0.0091	-2.0419	0.0420
DA	Data Analysis	0.00	0.0106	0.3883	0.6980
FL	Foreign Language	0.05	0.0094	4.5481	0.0000

Source: Authors' own creation

Table S9. Regression coefficients after confounding variable settings (learning rate of 0.01, 2000 iterations, 500 tests).

**Variable**	**Coefficient_Mean**	**Coefficient_Std**	**R_squared_Mean**	**R_squared_Std**
IL'	-0.02	0.00	0.02	0.00
RB'	-0.06	0.00	0.02	0.00
TU'	-0.08	0.00	0.02	0.00
PO'	0.04	0.00	0.02	0.00
CB'	-0.01	0.00	0.02	0.00
DA'	-0.08	0.00	0.02	0.00
FL'	0.03	0.00	0.02	0.00

Note: Table C3 corresponds to Figure 5 in section 4.4 of the manuscript

Source: Authors' own creation
